# Development and clinical validation of a robust knowledge‐based planning model for stereotactic body radiotherapy treatment of centrally located lung tumors

**DOI:** 10.1002/acm2.13120

**Published:** 2020-12-07

**Authors:** Justin Visak, Ronald C. McGarry, Marcus E. Randall, Damodar Pokhrel

**Affiliations:** ^1^ Medical Physics Graduate Program Department of Radiation Medicine University Kentucky Lexington KY USA

**Keywords:** adaptive re‐planning, centrally located lung SBRT, knowledge‐based planning, RapidPlan model

## Abstract

**Purpose:**

To develop a robust and adaptable knowledge‐based planning (KBP) model with commercially available RapidPlan^TM^ for early stage, centrally located non‐small‐cell lung tumors (NSCLC) treated with stereotactic body radiotherapy (SBRT) and improve a patient's“simulation to treatment” time.

**Methods:**

The KBP model was trained using 86 clinically treated high‐quality non‐coplanar volumetric modulated arc therapy (n‐VMAT) lung SBRT plans with delivered prescriptions of 50 or 55 Gy in 5 fractions. Another 20 independent clinical n‐VMAT plans were used for validation of the model. KBP and n‐VMAT plans were compared via Radiation Therapy Oncology Group (RTOG)–0813 protocol compliance criteria for conformity (CI), gradient index (GI), maximal dose 2 cm away from the target in any direction (D2cm), dose to organs‐at‐risk (OAR), treatment delivery efficiency, and accuracy. KBP plans were re‐optimized with larger calculation grid size (CGS) of 2.5 mm to assess feasibility of rapid adaptive re‐planning.

**Results:**

Knowledge‐based plans were similar or better than n‐VMAT plans based on a range of target coverage and OAR metrics. Planning target volume (PTV) for validation cases was 30.5 ± 19.1 cc (range 7.0–71.7 cc). KBPs provided an average CI of 1.04 ± 0.04 (0.97–1.11) vs. n‐VMAT plan'saverage CI of 1.01 ± 0.04 (0.97–1.17) (*P* < 0.05) with slightly improved GI with KBPs (*P* < 0.05). D2cm was similar between the KBPs and n‐VMAT plans. KBPs provided lower lung V10Gy (*P* = 0.003), V20Gy (*P* = 0.007), and mean lung dose (*P* < 0.001). KBPs had overall better sparing of OAR at the minimal increased of average total monitor units and beam‐on time by 460 (*P* < 0.05) and 19.2 s, respectively. Quality assurance phantom measurement showed similar treatment delivery accuracy. Utilizing a CGS of 2.5 mm in the final optimization improved planning time (mean, 5 min) with minimal or no cost to the plan quality.

**Conclusion:**

The RTOG‐compliant adaptable RapidPlan model for early stage SBRT treatment of centrally located lung tumors was developed. All plans met RTOG dosimetric requirements in less than 30 min of planning time, potentially offering shorter “simulation to treatment” times. OAR sparing via KBPs may permit tumorcidal dose escalation with minimal penalties. Same day adaptive re‐planning is plausible with a 2.5‐mm CGS optimizer setting.

## 1 INTRODUCTION

Stereotactic body radiotherapy (SBRT) for early stage localized non‐small cell lung cancer (NSCLC) has become a significant treatment option to traditional surgical intervention providing primary tumor local control rates in excess of 97% (median, 3 year).^1^, ^2^ Historically, lung SBRT was delivered using 7–13 co/non‐coplanar static beams or dynamic conformal arcs (DCA), followed by intensity modulation radiation therapy (IMRT) and more recently with volumetric modulated arc therapy (VMAT).^1^, ^3^, ^4^ VMAT provides more conformal dose distribution to the target better sparing of organs‐at‐risk (OAR) and much faster treatment delivery. The dosimetric advantages of VMAT can be enhanced using 6MV‐flattening filter free (6MV‐FFF) beam for lung SBRT because of its higher dose rates and reduction of out‐of‐target dose with respect to traditional flattened beams.^5^ This provides clinical benefits to the patients as it improves target coverage at the lung–tumor interface and shorter treatment time; potentially improving patient convenience and reducing intrafraction motion errors.^6^ In North America, the Radiation Therapy Oncology Group (RTOG) reports provides recommendations to clinicians for SBRT dosing schemata and contouring guidelines based on operable eligibility and tumor geographical location. This study concentrates on SBRT for early stage NSCLC patients with centrally located tumors following RTOG‐0813 guidelines.^7^ In addition to centrally located lung tumors, our clinic uses this protocol for risk‐adapted prescriptions for tumors located adjacent to critical structures such as the ribs.

Generating an optimal SBRT treatment using a VMAT approach requires multiple iterations and heavily depends on a planner'sskill. This potentially results in inconsistent plan quality known as interplanner variability.^8^, ^9^ Automation of inverse planning via knowledge‐based planning (KBP) aims to remove interplanner variability, improve plan quality, and decrease planning time.^10^ KBP uses a model library of previously generated high‐quality clinical plans to predict new treatment parameters, effectively generating new plans based on a clinic'streatment planning history.^11^ A Varian RapidPlan (Varian Medical Systems, Palo Alto, CA, USA) model is a KBP engine that utilizes a knowledge‐based dose–volume histogram (DVH) algorithm to estimate the DVH that can produce optimization objectives such as maximum, minimum, and new line dose constraints with associated priority values.^12^ KBP has demonstrated the ability to create improved or equivalent plans for prostate, head and neck, spine, breast and thoracic sites.^8^, ^13^, ^14^, ^15^, ^16^, ^17^, ^18^ However, there is very limited literature available for lung SBRT treatments,^14^, ^15^, ^17^specifically utilizing highly conformal non‐coplanar VMAT (n‐VMAT) planning geometry.

In this report, a RapidPlan model is described to generate adaptable n‐VMAT‐based KBP treatment plans for early stage NSCLC patients with medically inoperable centrally located tumors that follows RTOG‐0813 dosing schemata and contouring guidelines. Our model is exclusively trained with clinically treated high‐quality n‐VMAT lung SBRT plans using the advanced AcurosXB final dose calculation algorithm. We use the advanced AcurosXB algorithm for heterogeneity corrections for lung SBRT treatments as it provides a more accurate dose calculation in heterogeneous patient anatomy by better modeling secondary build‐up in tissue/low‐density interfaces than traditionally used superposition/convolution algorithms.^19^, ^20^ The KBP model may permit the improvement of “simulation to treatment” time from our current average 7 working days to 3 days while maintaining plan consistency and reducing interplanner variability. This may enable same or next day adaptive treatments (if needed) that aim to account for day‐to‐day changes in physiological characteristics or setup errors as they occur during a treatment course. A previous study using a smaller calculation grid size (CGS) of 1.25 mm vs. 2.5 mm in manually optimized VMAT lung SBRT plans with the photon optimizer (PO) algorithm demonstrated minimal dosimetric differences between the two plans but has not yet been evaluated in a lung SBRT KBP setting.^21^ This led to further evaluation of the concept by generating KBPs with a CGS of 2.5 mm which drastically decreases treatment planning time (mean, 5 min) and observe if they provide similar plan quality to the KBPs plans optimized with a 1.25‐mm CGS.

## 2 MATERIALS AND METHODS

### 2.1 Patient population and target definition

Following approval from our Institutional Review Board (IRB), 106 clinically treated high‐quality n‐VMAT lung SBRT plans generated for patients with early stage centrally located tumors as defined by RTOG‐0813 were selected for training and validation. Eighty‐six plans were used for training this model and the remaining 20 were used for validation. Patients received a total of 50 Gy or 55 Gy in 5 fractions. Details of the patient setup and simulation are published in detail elsewhere.^6^ Motion control of the target lesion was accomplished primarily by abdominal compression. If a patient had a contraindication to abdominal compression, for example, abdominal aortic aneurysm, extensive abdominal surgery, etc., a four‐dimensional (4D) CT simulation was done to create an internal target volume (ITV). For patients with abdominal compression, the gross tumor volume (GTV) was contoured on lung windows and a planning target volume (PTV) was added with margins of 1.0 cm superior/inferior and 0.5 cm laterally. For patients with 4D CT planning, an ITV was created from the maximum intensity projections (MIP) on lung windows and a uniform 0.5‐cm PTV margin was added uniformly per RTOG 0813 requirements. No clinical target volume (CTV) was allowed. OARs such as spinal cord, ipsilateral brachial plexus, skin, esophagus, heart, trachea, total lungs minus PTV, ribs, and bronchial tree were delineated per RTOG‐0813 compliance criteria for dose tracking.

### 2.2 Clinical n‐VMAT plans

For all patients, n‐VMAT SBRT plans were generated in the Eclipse treatment planning system (Varian Medical Systems, Palo Alto CA) using 3–6 (mean, 4) partial non‐coplanar arcs (with ±5°–12° couch kicks) on Truebeam Linac (Varian Palo Alto, CA) consisting of standard millennium 120 MLC and 6MV‐FFF (1400MU/min) beam. Jaw tracking option was enabled for each arc and optimal collimator angles were selected to minimize non‐target dose and enhance plan conformity. Clinical plans were optimized using Photon Optimizer (v13.6 or v15.6) algorithm with either 1.25‐mm or 2.5‐mm voxel resolution. The final dose calculation was performed using the advanced AcurosXB algorithm with dose to medium reporting mode. A dose of 50 Gy or 55 Gy in five treatments was prescribed to cover at least 95% of the PTV receiving 100% of the prescribed dose ensuring that all hotspots were within the PTV. Before approval, each plan was rigorously evaluated by our treating physicians via RTOG‐0813 compliance criteria and institutional guidelines including dose to OAR listed below:

1. Conformity index (CI): ratio of 100% isodose line volume to PTV volume, typically 1.0 < CI < 1.2.
2. Gradient index (GI): ratio of 50% isodose line volume to PTV volume, typically 3.0 < GI < 6.0 based on tumor size
3. D2cm (%): maximum dose in any direction 2 cm away from the PTV, typically 50% < D2cm < 70% based on tumor size.
4. Gradient distance (GD): average distance from 100% isodose line to 50% isodose line, indicator of intermediate dose spillage and sharp fall‐off.
5. Total monitor units (MU).
6. Modulation factor (MF): total number of monitor units divided by the prescription dose in cGy.
7. Beam‐on time (BOT).
8. Dose to OAR: Maximal and volumetric dose to OAR.

### 2.3 KBP model input and training datasets

**FIG. 1:**
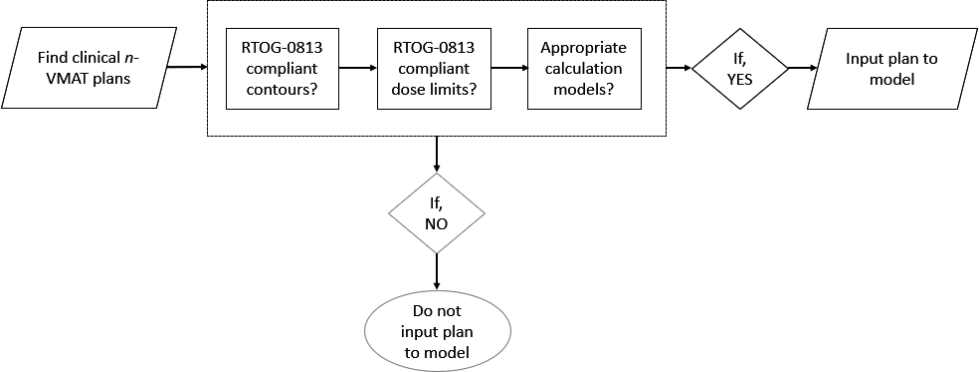
KBP‐model training input data selection workflow for centrally located lung SBRT: A total of 86 high‐quality clinical n‐VMAT plans were selected to train this model that met RTOG‐0813 requirements for contouring and OAR dose tolerances while using Acuros‐based dose calculation.

An extensive iterative training approach was developed to create this novel and comprehensive KBP model for SBRT of centrally located lung tumors. Eighty‐six n‐VMAT plans were retrospectively selected and verified to be high quality by evaluating the numbers of partial arcs and total MU consistency based on historical treatment planning practice. Original (unaltered) clinical VMAT plans were used for model training. The primary focus of this plan selection process was examination of RTOG‐0813 criteria. Each plan contour was individually verified to be consistent and correct. A total lung minus PTV structure was added for each patient'splan if the structure was not previously created. Calculation models consisting of dose calculation algorithm, VMAT MLC optimizer and CGS were verified to be AcurosXB for a 2.5‐mm resolution voxel size and photon optimizer for a 1.25‐mm or 2.5‐mm voxel size, respectively. Optimal collimator angle and jaw tracking options were verified prior to input of the training plans. To make the model fully comprehensive for RTOG compliance, it was necessary to track and select plans of varying target size and tumor geographical locations (e.g., lower lobe vs. upper lobe, right lung vs. left lung) encompassing the both lungs (see Table 2). Figure 1 shows a summarized workflow of initial plan selection criteria.

### 2.4 Verification of the KBP model

**FIG. 2:**
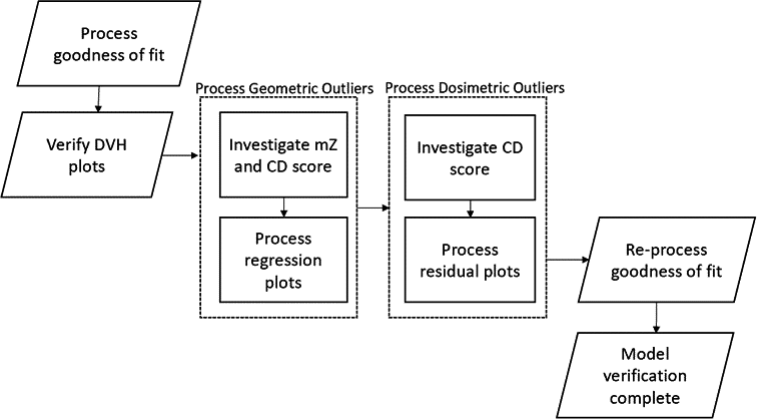
KBP model training workflow: The model was trained by locating and removing the geometric and dosimetric outliers iteratively.

Verification of a model is a process to evaluate the goodness of fit of the model to ensure proper generation of each OAR DVH estimate. Model verification was accomplished by using data provided by the RapidPlan engine to evaluate the R^2^ fitting values and chi‐squared values for each DVH estimate provided in the model‐training log. If these values are suboptimal, this is due to the presence of outlier plans in the model. There are two different types of outliers in the plans: dosimetric and geometric.^14^ The RapidPlan engine aids in the removal of outliers; for each OAR, it provides in‐field DVH plots, geometric box plots, principal component analysis‐regression, and residual plots coupled with a window of different statistics used to gauge a plan'squality of fit into a model. The provided regression and residual plots were evaluated for each OAR were used for manual verification of potential outliers.^22^ This approach was combined with observing the Cook'sdistance that indicated influential data points in a regression model and the modified Z‐score, which measures the difference of an individual geometric parameter from the median value in the training set.^23^ Once true outliers were identified; the entire plan or specific outlying structure was removed from the model and all data was re‐extracted. A summary of the KBP model refinement process is shown in Fig. 2.

Constraints were placed on a given OAR following successful verification of the model to create a fully robust model for centrally located lung tumors and risk adapted tumor location such as those tumors abutting the rib (see Table 1). Theses constraints were chosen based on RTOG‐0813 guidelines and our historical treatment planning practice.

**TABLE 1:** Selected constraints and their priority for the OAR used to generate the KBP model.

Structure	Constraint type	Vol (%)	Dose	Priority
Brachial plexus	Upper	5.0	2360 cGy	Gen.
	Upper	0	2720 cGy	Gen.
Bronchial Tree	Upper	0.0	105%	Gen.
	Line (prefer target)	Gen.	Gen.	Gen.
Spinal cord	Upper	0	2100 cGy	
	Upper (fixed volume, gen dose)	2.0	Gen.	Gen.
	Line	Gen.	Gen.	Gen.
D2cm	Upper	0.0	50%	110
	Line (prefer target)	Gen.	Gen.	Gen.
Esophagus	Upper	0	105%	Gen.
	Line (prefer OAR)	Gen.	Gen.	Gen.
Heart	Upper	0.0	105%	Gen.
	Line (prefer target)	Gen.	Gen.	Gen.
Ribs	Upper	0	4000 cGy	Gen.
	Upper (fixed dose, gen vol.)	Gen.	3200 cGy	Gen.
	Line (prefer target)	Gen.	Gen.	Gen.
Skin	Line (prefer target)	Gen.	Gen.	Gen.
Trachea	Line (prefer target)	Gen.	Gen.	Gen.

Gen., Generated; OAR, organs‐at‐risk; KBP, knowledge‐based planning.

### 2.5 Validation of the KBP model

A total of 20 clinical n‐VMAT plans that were not used to generate the RapidPlan model were selected for final verification including recently treated lung SBRT patients where dedicated manual planning time was recorded (Table 2). These plans were specifically selected to encompass both lungs’ geometry and variable target sizes to fully test the functionally of our model'srobustness. However, plan quality was not evaluated prior to selection to ensure the model could produce optimal plans for various case complexities. The overall validation set included 16 patients who received 50 Gy and 4 patients who received 55 Gy in 5 fractions, respectively. These plans were re‐optimized with the RapidPlan model with identical planning geometry as the clinical n‐VMAT plans. KBPs were created from a single optimization with no manual intervention. Target dose coverage for the KBPs was normalized for identical or better target coverage compared to previously used clinical n‐VMAT plans.

**TABLE 2:** Patient cohort and tumor characteristics for both training and validation of this comprehensive RTOG‐compliant KBP model. Overall, patient cohort and respective tumor geographical location including tumor size mean ± SD (range) are presented.

Tumor location	Training set		Validation set	
	Patients	PTV (cc)	Patients	PTV (cc)
Overall cohort	n = 86	35.7 ± 26.7 (4.4–158.3)	n = 20	30.5 ± 19.1 (7.0–71.7)
Right lower lobe (RLL)	n = 23	42.9 ± 35.2 (10.4–158.3)	n = 5	29.4 ± 19.8 (7.5–58.9)
Right upper lobe (RUL)	n = 30	29.1 ± 20.1 (4.4–78.7)	n = 6	30.3 ± 23.8 (7.0–71.7)
Left lower lobe (LLL)	n = 16	34.1 ± 27 (9.4–105.3)	n = 4	24.1 ± 8.6 (12–33.1)
Left upper lobe (LUL)	n = 17	39.1 ± 19.2 (9.0–70.8)	n = 5	37.0 ± 16.1 (12.5–51.3)

N, no. of patients; KBP, knowledge‐based planning; RTOG, Radiation Therapy Oncology Group.

To fully assess the performance of this new KBP model, we evaluated the target conformity, dose‐fall off and intermediate dose spillage. Additionally, dose‐limiting criteria for organs such as spinal cord, skin, esophagus, trachea, heart, lungs minus PTV, ribs, and bronchial tree were evaluated. A paired Student's*t*‐test (Microsoft Excel, Microsoft Corp., Redmond, WA, USA) was used to evaluate KBP vs. clinical n‐VMAT plans. Plan complexity was assessed by calculating MF. We also recorded the beam‐on time which is proportional to the changes in MF. Quality assurance phantom measurements of both n‐VMAT and KBPs were performed using an Octavius detector 1500 and phantom with 7.1 mm center‐to‐center detector spacing (PTW, Freiburg, Germany) to better assess the treatment delivery accuracy. KBPs were initially optimized using a 1.25‐mm CGS in the PO MLC algorithm configuration. To assess the feasibility of using this KBP model for the same day adaptive re‐planning, KBPs were re‐optimized with a 2.5‐mm CGS. Plan quality and re‐optimization time were assessed by comparing to the original KBPs plans.

## 3 RESULTS

### 3.1 Dosimetric criteria

Knowledge‐based plans were able to provide similar or better target coverage than clinical n‐VMAT plans (Table 3). KBPs had a slightly higher conformity index of 0.03 (*P* < 0.05) on average, indicating better overall target coverage than n‐VMAT plans including enhancing minimum dose to GTV. The gradient index was on average lower by 0.28 for KBP (*P* < 0.05) suggesting the KBP model was able to provide a more homogenous dose to the target with sharper and lower intermediate dose spillage outside the target. While a difference in D2cm was not observed to be statistically significant, there was a lower difference in the gradient distance (*P* < 0.05) suggesting KBPs had a sharper 50% isodose fall off.

**TABLE 3:** Evaluation of the conformity index and gradient indices for all 20‐lung SBRT patients that were generated via the KBP model for validation. Mean value ± SD (range) and p‐values were reported.

Target	Parameter	KBP	n‐VMAT	*P*‐value
PTV	CI	1.04 ± 0.04 (0.97–1.11)	1.01 ± 0.04 (0.97–1.17)	**0.002**
	GI	4.12 ± 0.9 (3.10–6.53)	4.40 ± 0.7 (3.39–6.01)	**0.003**
	HI	1.25 ± 0.05 (1.15–1.35)	1.24 ± 0.06 (1.16–1.39)	n. s.
	D_2cm_ (%)	51.2 ± 0.4 (0.41–0.57)	50.2 ± 0.4 (44.6–61.6)	n. s.
	GD (cm)	1.01 ± 0.2 (0.72–1.35)	1.11 ± 0.2 (0.78–1.62)	**<0.001**
	D99% (Gy)	49.1 ± 2.2 (46.6–54.5)	49.6 ± 2.0 (47.4–53.9)	**0.004**
	Mean (Gy)	57.1 ± 2.4 (54.4–62.4)	55.8 ± 2.4 (52.7–61.4)	**0.003**
	Maximum (Gy)	62.1 ± 3.0 (58.1–69.5)	62.3 ± 2.8 (57.2–67.5)	n. s.
GTV	Minimum (Gy)	56.0 ± 3.1 (50.8–62.6)	54.9 ± 3.3 (50.1–61.9)	**0.05**
	Mean (Gy)	59.6 ± 2.5 (56.2–65.6)	59.1 ± 2.8 (55.4–65.8)	n. s.

n. s., not significant; KBP, knowledge‐based planning; SBRT, stereotactic body radiotherapy.

Significant values are highlighted in bold.

**FIG. 3:**
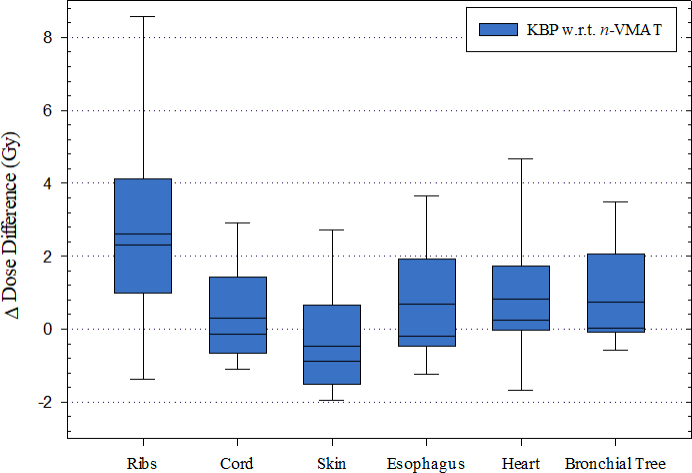
Box plot of maximal pairwise dose differences of KBP compared to n‐VMAT plans displaying median, 10th, 25th, 75th, and 90th percentiles with error bars. Negative values indicate that KBPs provided less sparing relative to n‐VMAT plans. All 20 lung SBRT cases used for validation were included. Prescription was 50 or 55 Gy in 5 fractions. The KBP model was able to spare maximum rib dose on average by 2.62 Gy (maximum up to 9.67 Gy). Maximum skin dose was on average higher by 0.46 Gy (*P* = n. s.) but not clinically significant in KBPs.

Dose to normal lung was tracked using mean lung dose, and the volume receiving 5 Gy (V5) 10 Gy (V10), 20 Gy (V20), or more. These results are shown in Table 4. KBPs had an average lower V5Gy by 0.6%, (*P* < 0.001), V10Gy by 0.5% (*P* < 0.001), and MLD by 0.12 Gy (*P* < 0.001) suggesting a potentially lower risk of radiation‐induced pneumonitis. In addition to normal lung tissue doses, all other OAR compliance criteria were assessed per RTOG‐0813 (Fig. 3). In many lung SBRT cases, risk‐adapted prescription to targets adjacent to the ribs are used. The greatest sparing achieved in the KBPs was shown in the ribs (*P* < 0.001) for an average of 2.62 Gy (maximum up to 9.67 Gy).

**TABLE 4:** Evaluation of the dosimetric lung data for all 20 lung SBRT validation cases. Mean value ± SD (range) and p‐values were reported.

DVH Parameter	KBP	n‐VMAT	*P*‐value
V5Gy (%)	10.7 ± 5.1 (3.4–19.7)	11.3 ± 5.2 (3.2–21.0)	**<0.001**
V10Gy (%)	6.6 ± 3.8 (2.4–14.1)	7.1 ± 4.1 (2.3–15.4)	**<0.001**
V20Gy (%)	2.7 ± 1.8 (0.7–6.7)	2.8 ± 1.9 (0.8–7.7)	**0.007**
MLD (Gy)	2.29 ± 1.2 (0.95–4.9)	2.41 ± 1.2 (0.8–5.2)	**<0.001**

MLD, mean lung dose; V5, V10, V20, volume of lung receiving 5 Gy, 10 Gy, 20 Gy, or more, respectively. n. s., statistically not significant; SBRT, stereotactic body radiotherapy; DVH, dose–volume histogram.

Statistically significant values are highlighted in bold.

Our study showed that the ipsilateral brachial plexus, esophagus, heart, trachea, and bronchial tree received an insignificant average lower dose in KBPs compared to the clinical n‐VMAT plans. Additionally, KBPs on average presented an insignificant but slightly higher skin dose to spinal cord by 0.46 Gy (*P* = 0.32).

### 3.2 Treatment planning time, delivery efficiency, and accuracy

Knowledge‐based plans were generated and ready for treatment plan review on average in under 30 min, providing a clinically relevant reduction in treatment planning time. For an experienced planner with dedicated SBRT planning time, manual plans were created in 129 ± 34 min, on average (range, 95–183 min). Table 5 displays treatment and delivery efficiency metrics for KBPs and n‐VMAT plans. KBPs on average only increased total monitor units by 460 (*P* = 0.008). When considering nominal maximal dose rates of 6MV‐FFF beam (1400MU/min), this results in similar beam on time. The minimal values of MF and BOT were increased by 0.46 (*P* = 0.008), and 19.2 s (*P* = 0.008), respectively. However, KBPs were still able to provide enhanced GTV dose and lower dose to OAR.

**TABLE 5:** Treatment delivery efficiency and accuracy of KBP with respect to clinical n‐VMAT plans. Mean value ± SD (range) and p‐values were reported for both KBP and n‐VMAT plans.

Treatment delivery parameter	KBP	n‐VMAT	*P*‐value
Total monitor units	3480 ± 531 (2553–4639)	3020 ± 674 (1961–4104)	**0.008**
Modulation factor	3.48 ± 0.53 (2.53–4.64)	3.02 ± 0.67 (1.92–4.10)	**0.008**
Beam‐on time (min)	2.49 ± 0.34 (1.81–3.31)	2.15 ± 0.48 (1.37–2.93)	**0.008**
γ‐pass rate (2%/2mm)	94.4 ± 2.7 (90.6–100.0)	95.4 ± 2.3 (90.9–99.4)	0.11

n. s., statistically not significant; KBP, knowledge‐based planning; n‐VMAT, non‐coplanar volumetric modulated arc therapy.

Significant values are highlighted in bold.

In the patient‐specific quality assurance measurements, the gamma analysis of 2%/2 mm criteria was used to assess the plan delivery accuracy differences between KBP vs. clinical n‐VMAT plans. KBPs presented with a similar average pass rates of 94.4 ± 2.7% (range, 90.6–100.0%) compared to n‐VMAT plans with an average pass rates of 95.4 ± 2.3% (range, 90.9–99.4%) (*P* = 0.11) plans suggesting that comparable treatment delivery accuracy can be achieved with KBPs.

### 3.3 Example validation case – Left lower lobe tumor

**FIG. 4:**
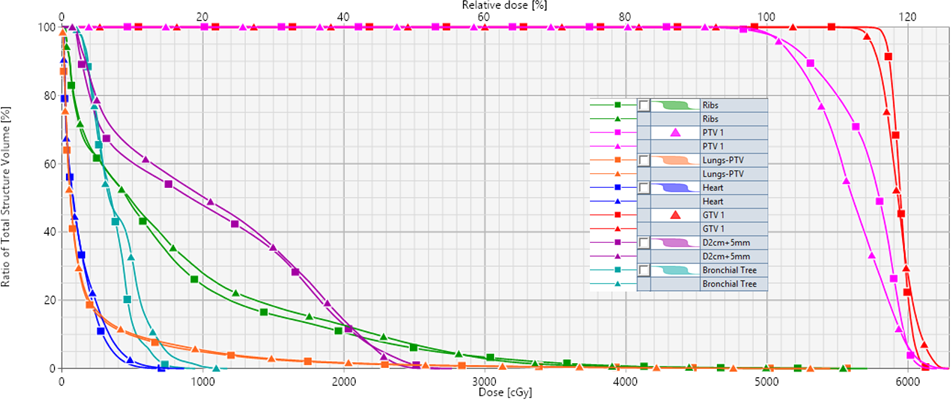
Dose–volume histogram comparison for the target coverage for the GTV (red) and OAR such as total normal lung minus PTV (orange), heart (dark blue), ribs (green), and bronchial tree (dark blue) are shown for an example patient KBP plan (square), and n‐VMAT (triangle). Prescription dose was 50 Gy in 5 fractions. KBP provided superior target coverage and lower dose to the OAR.

**FIG. 5:**
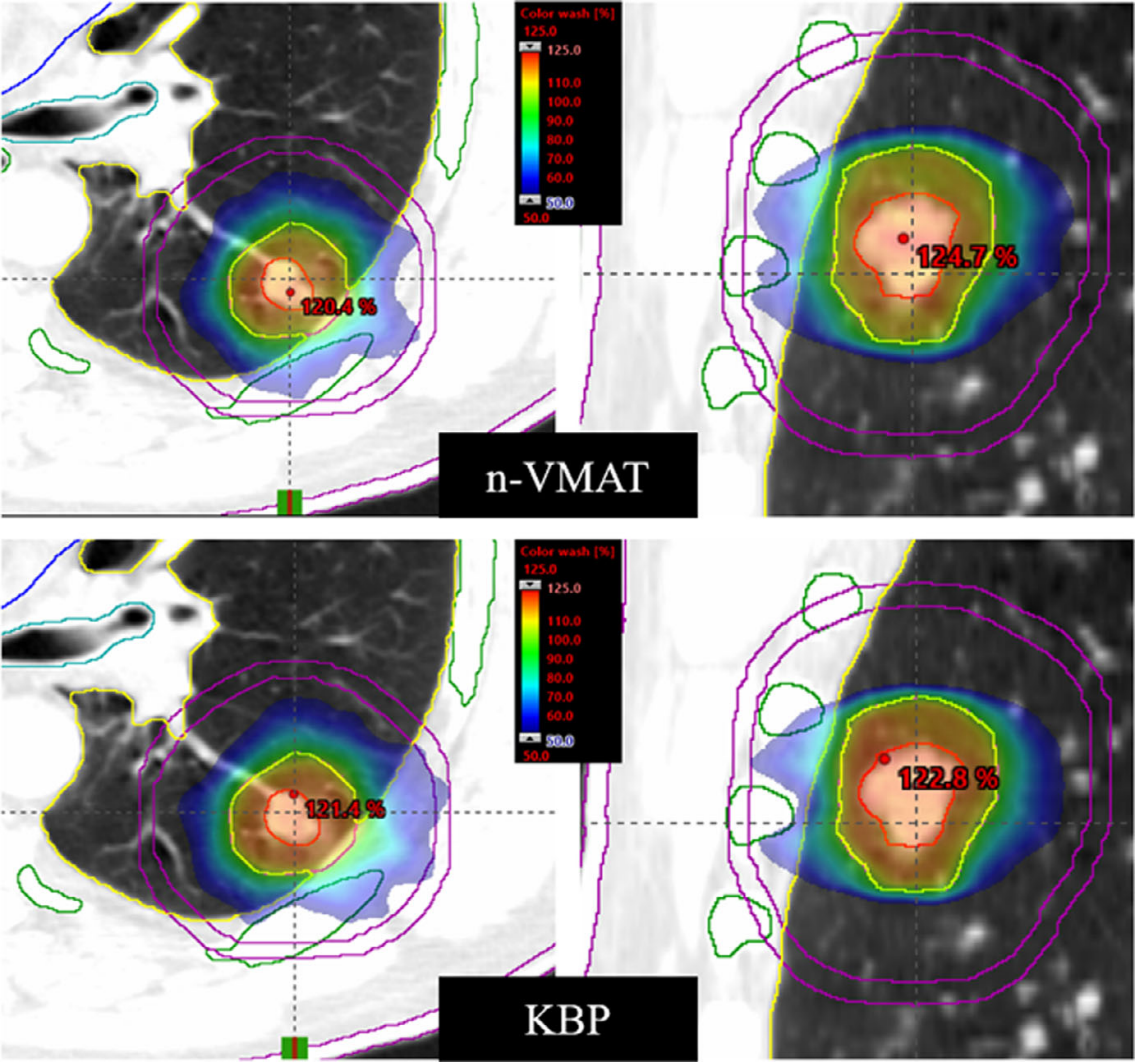
Comparison of KBP vs a clinical n‐VMAT plan for the example validation case. The axial and coronal views of SBRT isodose distributions for the clinical n‐VMAT plan (upper panel) and the corresponding KBP plan (lower panel) are shown. Tumor was located in the left lower lobe and treated for 50 Gy in 5 fractions. Similar, CI, GI, D2cm, GD, and V_20Gy_ were obtained. A few critical structures shown were ribs, skin, bronchial tree, ipsilateral normal lung, as well as D2cm ring (purple contour). Tighter 50% dose colorwash showing lower rib dose providing slightly better target coverage with KBP plan.

Dose–volume histograms of both the KBP and n‐VMAT plan for a validation case with a left lower lobe tumor of a lung SBRT patient were generated (Fig. 4). This patient was selected as the example case as it best represented the average expectation of improvement using the KBP model. With better target coverage (minimum dose to GTV was increased by 2.3 Gy), the KBP model was able to lower volumetric dose to lungs including MLD, ribs, heart, and bronchial tree. In this case, the maximum rib dose was reduced by 4.2 Gy compared to clinical n‐VMAT plan. Both plans were normalized so at least 95% of PTV received 100% of the prescribed dose. The dosimetrically superior plan was generated using the KBP model, as demonstrated with slightly better target coverage and volumetrically lower dose to the OAR including lower maximal dose to rib (Fig. 5).

### 3.4 Re‐optimized KBPs with 2.5 mm CGS

The KBP calculation time was dictated by the CGS used in the optimization window. The original KBPs were calculated with a 1.25‐mm CGS. However, while using a 2.5‐mm CGS the treatment planning time was reduced to approximately 5 min. This setting could support even faster adaptive re‐planning in emergent clinical situations. Therefore, KBPs were re‐optimized with a 2.5‐mm CGS for plan evaluation. Table 6 displays sample target coverage and normal tissue sparing dose volume histogram metrics. It was found that these plans could be created in 5 min, on average, with minimal loss of dosimetric plan quality. Conformity index and gradient index showed no statistical difference between 1.25‐ vs. 2.5‐mm CGS re‐optimized plans. Therefore, KBPs indicate similar conformal and homogenous target dose coverage with a 2.5‐mm CGS. Gradient distance (*P* = 0.45) was slightly increased with a 2.5‐mm CGS configured KBPs signifying an increase in intermediate dose spillage, however, clinically acceptable and similar V20Gy values were observed (Table 6).

**TABLE 6:** Selected target coverage and DVH parameters for re‐optimized KBPs with a 2.5‐mm CGS vs original KBPs with an 1.25‐mm CGS. Average absolute difference ± SD (range) and *P*‐values were reported between the two plans.

	Average difference: 1.25 minus 2.5 mm CGS KBPs	*P*‐value
Plan metrics		
Conformity index	0.02 ± 0.02 (0.0–0.06)	0.46
Gradient index	0.32 ± 0.59 (0.0–2.4)	0.93
Gradient distance (cm)	0.03 ± 0.08 (0.0–0.19)	0.04
DVH Metrics		
V20Gy (%)	0.08 ± 0.07 (0.0–0.3)	0.53
Maximum rib dose (Gy)	0.57 ± 0.44 (0.8–1.5)	**<0.001**
Maximum cord dose (Gy)	0.41 ± 0.50 (0.0–2.33)	0.45

n. s., statistically not significant; KBP, knowledge‐based planning; DVH, dose–volume histogram; CGS, calculation grid size.

Significant values are highlighted in bold.

As shown in Table 6, our study found that maximum dose differences for the rib and spinal cord were not clinically significantly different (similar results were found for other OAR, not shown here) indicating that a 2.5‐mm CGS can be used for safe and effective adaptive re‐planning of lung SBRT cases (for selected patients) using this KBP model.

## 4 DISCUSSION

A fully RTOG‐0813‐compliant, n‐VMAT‐based KBP model using Varian RapidPlan was developed and validated for centrally located lung tumors treated with SBRT. This novel model was fully trained with high‐quality clinical plans that adhere to contouring, prescription schemata and dose limits set forth by RTOG‐0813 without prior alteration to input to the model. It is likely that any clinic that complies with RTOG protocol constraints and are RapidPlan capable can potentially adapt this model to provide high‐quality n‐VMAT lung SBRT treatments. To our best knowledge, this novel model is the first RapidPlan model created exclusively for centrally located tumors using a non‐coplanar VMAT approach with the more accurate Acuros‐based dose calculation engine. This comprehensive KBP model can encompass centrally located lung tumors as well as those near the ribs.

One of the major benefits of using this RapidPlan model is its possibility of improving clinic workflow of “simulation to treatment” time from 7 to 3 working days. While this study recorded an average dedicated planning time of approximately 129 min for an experienced planner, in our clinic, the majority of our standard 7 working day “simulation to treat time” comes from planning. We do not have dedicated SBRT planners and this standard time slot accounts for not only planner workload but also interplanner variability. Our institution'splanners simultaneously plan multiple treatments per day and do not have the time to meticulously optimize each lung SBRT plan unlike a dedicated planner. Additionally, patients who present for re‐treatment, have an implanted pacemaker, or any other unique planning difficulty can increase planning time up to a week. Therefore, the KBP model may allow adaptive re‐planning for selected patients with incorrect patient set‐up on the machine, weight loss or tumor shrinkage that will maintain high‐quality SBRT treatment delivery in a timely manner. As expected, for most tumors, this model can generate a plan of similar or better quality much faster than manual planning approach, while removing interplanner variability and standardizing the clinic workflow. This concept was expanded further by evaluating the effects of the photon optimizer CGS on a KBP model to evaluate the dosimetric trade‐off with decreased treatment planning time. This appears to be the first study evaluating CGS effects in the context of lung SBRT KBP planning. It is shown that by utilizing 2.5 mm CGS same day adaptive re‐planning is plausible as planning time was decreased to approximately 5 min with minimal dosimetric impact.

Moreover, the validation cases have shown that slight tumor dose escalation can be achieved in selected lung SBRT cases with similar plan quality to clinical plans and no penalty to dose‐limiting organs (DLO). For example, this KBP model can potentially reduce maximum dose to the rib by 2.62 Gy, on average, while also reducing dose in the ipsilateral brachial plexus, esophagus, heart, bronchial tree and trachea with no significant increases to other DLO like the spinal cord. Normal lung tissue dosing was also reduced in KBPs indicated by the reported V5Gy, V10Gy, V20Gy, and MLD. Again, these possible indicators of radiation‐induced pneumonitis.^23^, ^24^, ^25^ This dosimetric OAR sparing and slight dose escalation of tumor dose was achieved with minimal increase of plan complexity and overall beam on time. Plan deliverability and small field dosimetry errors were minimal as seen by similar quality assurance pass rates between the two plans. This indicates that the optimizer in the KBP model was not significantly modulating the treatment plans more to achieve better OAR sparing.

In the past, some investigators have generated KBP models for lung SBRT treatments.^14^, ^15^, ^17^ However, this model is different as it is the first to exclusively consider centrally located tumors, eliminating varied normal tissue DLO limits due to variable tumor location and prescriptions as seen in other models. For instance, this work differs from Chin *et al*. because they trained their model with a majority of IMRT plans with the less accurate analytical anisotropic algorithm (AAA) in their training datasets, resulting in different dosimetric sparing capabilities.^14^ They reported an average maximum dose increase to the esophagus of 1.1 Gy in their VMAT validation, whereas our KBP model reduced the maximum esophageal dose by 0.7 Gy, on average. Another study by Delaney *et al*. generated a lung SBRT model for peripheral lesions that considered both a 55 Gy in 5 fractions and 54 Gy in 3 fractions dosing schema.^15^ Using different prescriptions cause changes in normal tissue dose limits that can be detrimental to OAR sparing because of their different biological response to the organs. Our work differs from Delaney *et al*. as it fully covers centrally located tumors for a single prescription. The study by Hof *et al*. created a lung VMAT SBRT model to retroactively evaluate patients who devloped greater than grade 3 toxicities in tumors greater than 5 cm in diameter.^17^ They used a subset of their patients (tumors >5 cm) who did not develop toxicities as a training datasets. Due to lung toxicity, lung SBRT treatments are typically not done for tumors larger than 5 cm, so the KBP model described herein was designed for prosepective treatment of standard tumor sizes with centrally located lesions.

A limitation to our work (a common issue in other models) is that some patients’ geometries do not lend themselves to have a treatment ready lung SBRT plan in a single optimization. This limitation can be broadened to the idea that it is extremely difficult to create a KBP model that is fully robust. We found that in atypical cases treatment plans might need to be manually optimized further following automatic plan generation. While we feel that using 86 plans for training was sufficient, a few more atypical plans could be added to the model to better improve robustness of this model. However, there is also a risk of overfitting the model if too many plans are used for training the model. Future directions include adding more atypical cases into to further expand this model to tackle those extremely difficult cases. Our methods described in this work will be expanded next to generate and further validate a robust lung SBRT RapidPlan model for medically inoperable/operable early stage, peripherally located NSCLC patients using a recently developed dynamic conformal arc‐based VMAT planning method that further minimizes MLC complexity and improves SBRT treatment delivery efficiency and accuracy.^26^

## 5 CONCLUSION

This study created a lung SBRT KBP model via RapidPlan that can quickly generate a high‐quality n‐VMAT lung SBRT treatment plan for centrally located lung tumors per RTOG‐0813 protocol. This KBP model is fully comprehensive covering all ranges of tumor sizes and tumor geographical locations while maintaining adaptability for other clinics. Using this model, a lung SBRT treatment plan can be generated in less than 30 min, on average providing the ability to increase clinic workflow by reducing “simulation to treatment” time down to as few as 3 working days. This activates a clinic'sability for offline adaptive treatments to selected lung SBRT patients. Treatment planning time of KBPs was further reduced to 5 min while using PO 2.5‐mm CGS rather than 1.25 mm in the plan optimization without compromising plan quality. This supports same or next day adaptive re‐planning for selected lung SBRT patients. In addition to improving clinical workflow, our model was able to enhance hypoxic tumor core dose while better sparing critical structures compared to clinical VMAT plans. Moreover, it eliminates interplanner variability, benefiting standardizing lung SBRT treatment planning and improving patient safety. Clinical implementation of this KBP model will effectively improve overall clinic workflow and provide high quality, consistent, and highly conformal KBP lung SBRT treatments.

## CONFLICT OF INTEREST

The author have no other relevant conflicts of interest to disclose.

## AUTHOR'S CONTRIBUTIONS

DP and JV conceived the project. JV developed and validated the KBP model, and collected and analyzed the data. DP, RCM and MER provided clinical expertise and supervision of the paper. JV and DP drafted the manuscript and all co‐authors revised and approved the final manuscript.
